# SARS-CoV-2 occurrence in cervids in the United States and US territories

**DOI:** 10.1038/s41598-026-35967-8

**Published:** 2026-01-15

**Authors:** Sarah N. Bevins, Richard B. Chipman, Scott F. Beckerman, David L. Bergman, Derek T. Collins, Thomas J. Deliberto, Joshua P. Eckery, Jeremy W. Ellis, Allen L. Gosser, Jonathon D. Heale, Jason M. Klemm, Hannah Cluett, Kali Ward, Kristina Lantz, Timothy J. Linder, Mitch Oswald, Robert Pleszewski, Christopher A. Quintanal, Jourdan M. Ringenberg, Sean P. Streich, Kelsey R. Weir, Aaron T. Phillips, Bledar Bisha, Mia K. Torchetti, Julianna B. Lenoch, Jeffrey C. Chandler, Susan A. Shriner

**Affiliations:** 1 USDA/APHIS/WS National Wildlife Disease Program, Fort Collins, USA; 2 USDA/APHIS/WS National Rabies Management Program, Concord, USA; 3 USDA/APHIS/Wildlife Services, Riverdale, USA; 4 Department of Animal Science, University of Wyoming, Laramie, USA; 5 USDA/APHIS/WS National Wildlife Research Center, Fort Collins, USA; 6 USDA/APHIS/VS National Veterinary Services Laboratories, Ames, USA; 7 Western Association of Fish and Wildlife Agencies, Boise, USA

**Keywords:** SARS-CoV-2, Spillover, Wildlife, Zoology, Viral infection

## Abstract

SARS-CoV-2 was found in multiple cervid species throughout the United States from October 2021 through October 2023 and results reinforce findings of considerable viral spillover from people to cervids. Data demonstrate changes in viral and neutralizing antibody prevalence over time and as well as viral variants that differed from what was circulating in human populations. Continued monitoring can help pinpoint what is driving changes in prevalence and how those changes alter risk to both wildlife and human populations.

**Supplementary Information:**

The online version contains supplementary material available at 10.1038/s41598-026-35967-8.

## The study

There is now clear evidence that SARS-CoV-2 can move from humans to multiple other animal species^1,2^. This is especially true for white-tailed deer (*Odocoileus virginianus*) where early research found 40% of white-tailed deer in some regions had been exposed to SARS-CoV-2^3^ and more than one-third of deer in some populations were positive for the virus by rRT-PCR (real-time PCR with reverse transcription)^4^. This was an unexpectedly high level of infection for a human-to-deer spillover event of a pathogen that only recently emerged in people. Comparison of deer and human lineages suggests human-to-deer transmission is occurring repeatedly, followed by deer-to-deer transmission^5,6^. Recent data suggests that other cervid species can be infected as well^7^. Widespread persistence of the virus in multiple species exposes the pathogen to varying selection pressures, potentially leading to evolution of novel variants that could then be transmitted back to people^6,8–10^.

Given these concerns, a national-scale effort was initiated to monitor both viral infection and antibodies indicative of SARS-CoV-2 exposure in cervids across the United States (US) and US island territories^6,11^. Our goal with this broadscale surveillance was to highlight differences in infection and exposure across geographic regions and habitat types. In addition, capturing both virological prevalence and neutralizing antibody prevalence sheds light on both current and prior exposure, providing insight into SARS-CoV-2 dynamics.

## Methods

To achieve these monitoring goals, cervid samples were collected from 42 states, Washington DC, Guam, and the US Virgin Islands, from October 2021 through October 2023. Oral or nasal swabs preserved in PrimeStore Molecular Transport Media (MTM, Longhorn Vaccines and Diagnostics LLC), along with whole blood samples collected on Nobuto filter paper were obtained from cervids throughout the United States (white-tailed deer *n* = 30,529, mule deer (*Odocoileus hemionus*) *n* = 1539, moose (*Alces alces*) *n* = 251, axis deer (*Axis axis*) *n* = 159, Philippine deer (*Rusa marianna*) *n* = 159, elk (*Cervus canadensis*) *n* = 92, caribou (*Rangifer tarandus*) *n* = 79, Sitka black-tailed deer (*Odocoileus hemionus sitkensis*) *n* = 20). White-tailed deer were the focus of sampling because they were the only species with evidence of infection early on in the outbreak and because of their substantial overlap with human populations. Other species were added as evidence of infection beyond white-tailed deer became available. All sampling was carried out in accordance with relevant guidelines and regulations. Samples were collected in collaboration with hunters and state wildlife management agencies from hunter-harvested white-tailed deer, with additional samples coming from roadkill and deer being removed as part of approved and permitted wildlife management activities. Since this surveillance project primarily focused on opportunistic sampling of hunter-killed animals, additional animal research guidelines did not apply. Sampling was concentrated in fall and winter when hunter harvest activities typically occur, although roadkill and management samples were collected throughout the year. The goal was to collect both an oral swab and a blood sample from each animal sampled, although this was not always feasible, leading to variation in number of PCR verses serology results. Nonetheless, more than 30,000 of the 31,766 samples tested had a full set of samples.

SARS-CoV-2 RNA was extracted from oral and nasal swab samples using MagMAX™ CORE Nucleic Acid Purification Kits (Applied Biosystems) as previously described^12^ and in accordance with the manufacturer’s instructions. Singleplex rRT-PCR of SARS-CoV-2 N1 and N2 was performed using 5 µL of extracted RNA with the BioRad Reliance One-Step Supermix kit. SARS-CoV-2 Research Use Only qPCR Primers & Probes for the N1 and N2 regions of the viral genome, were provided by Integrated DNA Technologies. Thermal cycling conditions were 50 °C for 10 min and 95 °C for 10 min followed by 45 cycles of 95 °C for 10 s and 60 °C for 30 s using a BioRad CFX96 Touch Real-Time PCR Detection System or CFX Opus Real-Time PCR System. A positive designation required a SARS-CoV-2 N1 quantification cycle (Cq) < 38.5 in concert with an N2 Cq non-negative finding.

Viral sequencing and genome assembly were performed as previously described^6^. The Phylogenetic Assignment of Named Global Outbreak Lineages (PANGOLIN) software^13^ was used (PANGO v4.0.6 (2022-04-22)) to determine Pango lineages for each sequence.

Antibodies were extracted from Nobuto filter paper strips as described previously^3^. Briefly, Antibodies were extracted from Nobuto filter paper strips (Sterlitech, catalog # 49010010) and screened at the United States Department of Agriculture, National Wildlife Research Center (Fort Collins, USA) at a functional dilution of 1:20. Extracted samples were screened using a surrogate virus neutralization test (sVNT, Genscript cPass™, catalog# L00847-A and #A02161) with data acquired using a VarioSkan Flash or Varioskan LUX multimode microplate reader (Thermo Fisher). At least two technical replicates were used for the calculation of the average % inhibition. A subset of 19,733 samples from all cervid species where sample volume was sufficient were assayed for neutralizing antibodies using both the wildtype and Omicron sVNT assays. This was done to ensure antibody detections were robust even as variants changed over time.

Samples were considered positive if inhibition was ≥ 30% for the ancestral assay and > 40% for the Omicron assay^14^. The sVNT has not been validated for deer; however, previous evaluations with white-tailed deer sera samples suggested that sVNT results were qualitatively similar to a highly specific SARS-CoV-2 virus neutralization test. Blood samples on Nobuto filter paper were collected from all states, and in Ohio they were provided by the Ohio Animal SARS-CoV-2 Surveillance Consortium (Ohio State University Department of Veterinary Preventive Medicine, Ohio Department of Natural Resources Division of Wildlife, and US Department of Agriculture Wildlife Services).

All data supporting the findings of this study are available within the paper and its Supplementary Information. Viral sequence accessions deposited into GenBank (https://www.ncbi.nlm.nih.gov/genbank/) or GISAID (https://gisaid.org/).

## Results

**Fig. 1:**
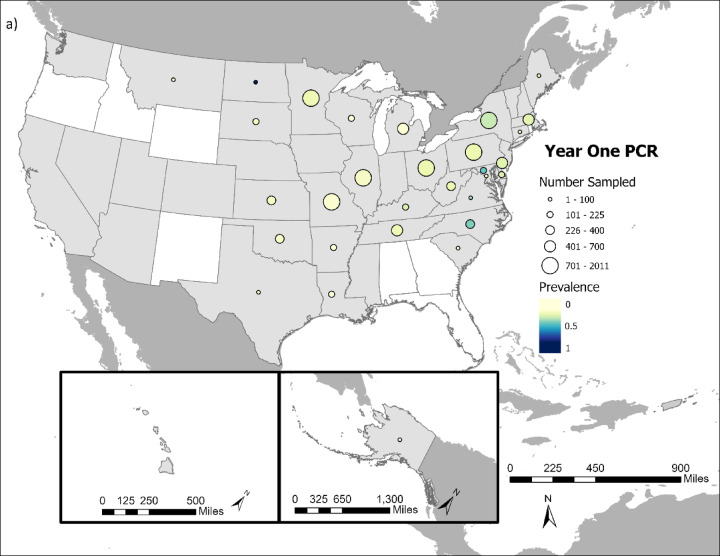

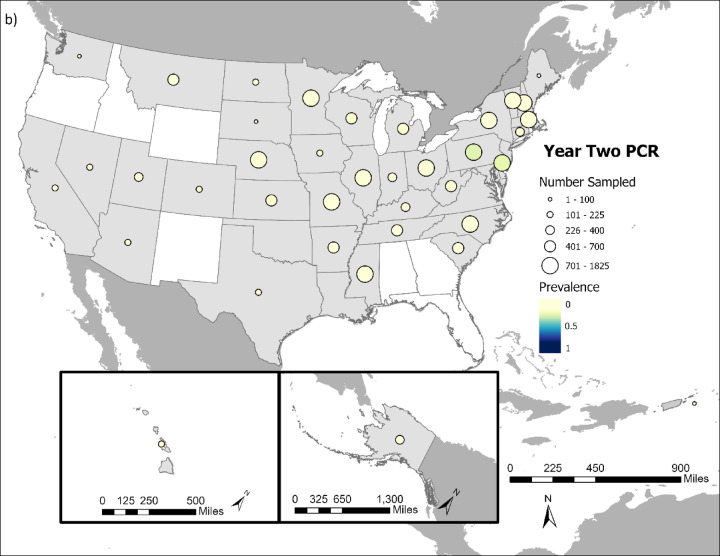

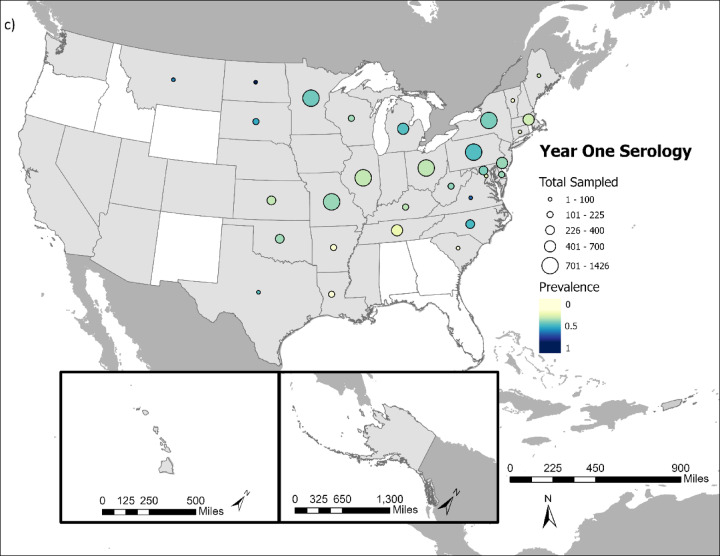

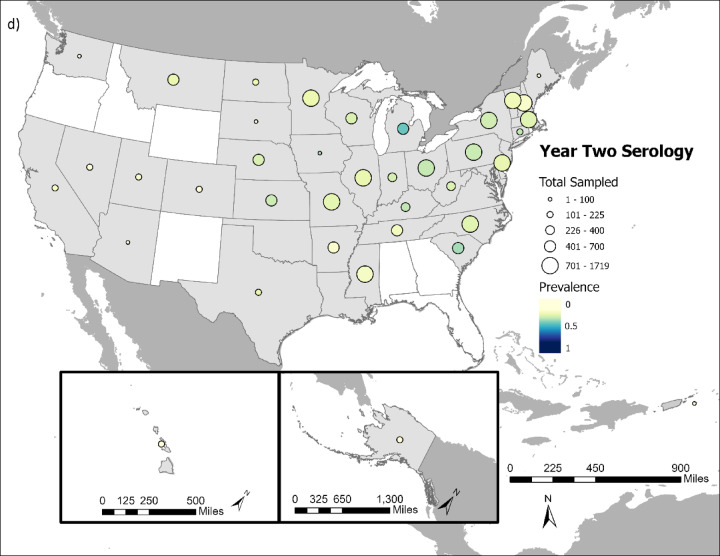
Comparison of cervid SARS-CoV-2 viral prevalence in (**a**) year 1 (2021/2022) and (**b**) year 2 of the study (2022/2023). Comparison of cervid SARS-CoV-2 neutralizing antibody prevalence in (**c**) year one (2021/2022) and (**d**) year 2 of the study (2022/2023). Circle size indicates the relative number of samples tested and color intensity indicates the relative rRT-PCR prevalence and neutralizing antibody prevalence within a state. States in white had no samples collected during course of study.

Overall, SARS-CoV-2 was detected in 5.58% (1,774 out of 31,766 tested) of cervids sampled (Fig. 1a and b), and 21.43% (6,477 out of 30,231 tested) had neutralizing antibodies indicative of SARS-CoV-2 exposure (Fig. 1c and d).

Most SARS-CoV-2 detections and neutralizing antibody detections were in white-tailed deer (1,770 PCR detections; 6,443 neutralizing antibody detections), although SARS-CoV-2 was also detected in mule deer (*n* = 4). SARS-CoV-2 neutralizing antibodies were also detected in mule deer (*n* = 30), moose (*n* = 3), and Philippine deer (*n* = 1).

**Fig. 2:**
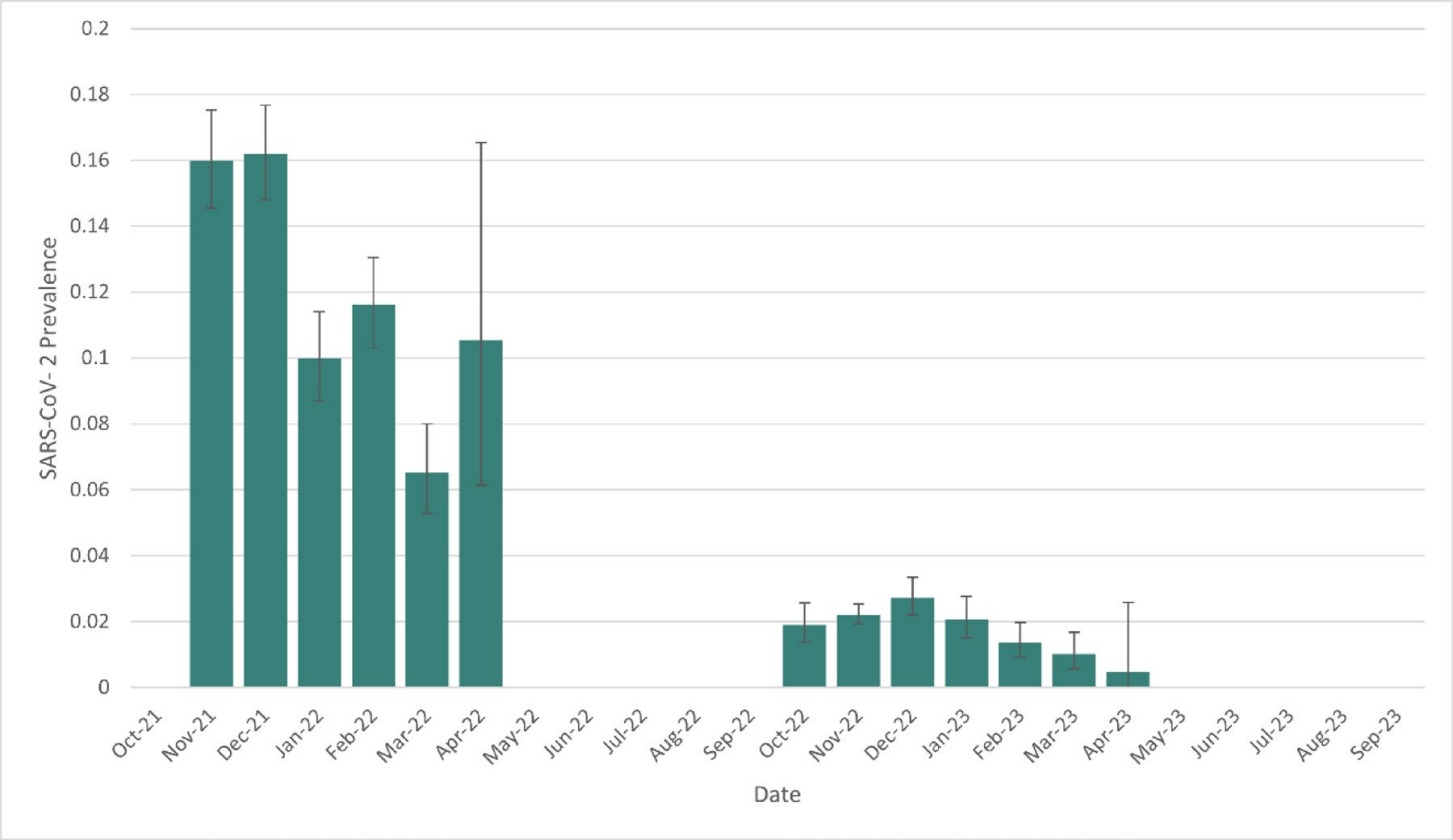
SARS-Cov-2 infection prevalence and binomial confidence intervals in white-tailed deer throughout the course of the study.

SARS-CoV-2 prevalence and neutralizing antibody prevalence were substantially higher during the first year of the study, which ran from October 1, 2021 – September 30, 2022 compared to the second year of the study, which ran from October 1, 2022-September 20, 2023 (Fig. 2). Year 1 viral prevalence was 12.27% while year 2 prevalence was 2.03%. Year 1 neutralizing antibody prevalence was 31.72% while year 2 prevalence was 16.06%. The significantly lower viral prevalence and seroprevalence in year 2 compared to year 1 occurred despite substantially more cervids being sampled and tested in year 2 (*n* = 21,140) versus year 1 (*n* = 11,431).

sVNT assays for Omicron and ancestral variants resulted in a prevalence of 17.34% (*n* = 3,421). The Omicron sVNT assay had 265 positive detections when the ancestral sVNT assay result was negative. These 265 samples resulted in a 1.34% increase in prevalence.

Analysis of white-tailed deer that were assayed by both rRT-PCR and sVNT revealed that 19.95% (5,635/27,747) were rRT-PCR negative but had neutralizing antibodies detected, an indication of widespread prior infection^3^. In addition, 2.87% (795/27,747) were rRT-PCR positive, but without detectable neutralizing antibodies, possibly indicating a recent or first-time exposure. 2.78% (772/27,747) were rRT-PCR positive and had neutralizing antibodies detected. 17,900 had results suitable for analysis for both ancestral and Omicron sVNT. 3,131 tested positive for ancestral sVNT assay, 1161 tested positive for Omicron sVNT assay, and 917 tested positive in both assays.

Genetic lineage classification was achieved for 354 sequences during year 1 of the study and Delta was the most common variant identified (273/354, 77% of variants identified), (Table 1), even though Delta was nearly completely replaced by Omicron in human populations^15–17^ by February of 2022, which was early on during the course of this study. Alpha was the second most commonly identified variant during year 1, followed by Gamma (Table 1). Omicron was only identified in 2/353 SARS-CoV-2 sequences from deer in year 1. During year 2, genetic lineage classification was achieved for 99 sequences, reflecting the significantly lower viral prevalence during this time period. For those lineages that were identified in year 2, Omicron was the most commonly identified variant (52/99), followed by Alpha (33/99), and Delta (14/99, Table 1).

**Table 1:** Number of SARS-CoV-2 variants identified from rRT-PCR positive samples.

Variant	Number of sequences identified 2021–2022	Number of sequences identified 2022–2023
Alpha	70	33
Delta	273	14
Gamma	9	0
Omicron	2	52

## Discussion

These broad-scale monitoring data show SARS-CoV-2 occurrence in cervids. White-tailed deer were the primary focus of this study and they were infected throughout the majority of their US range, reinforcing findings of considerable SARS-CoV-2 spillover to white-tailed deer.

While SARS-CoV-2 in white-tailed deer was widespread, findings also show a decrease in viral prevalence and neutralizing antibody prevalence over the course of the study. The overall viral prevalence reported here (5.58%) is also lower than smaller-scale white-tailed deer studies completed recently^4,5,8,18^. These differences may reflect deer populations gaining humoral protection from prior exposure to SARS-CoV-2. This is supported by the 80% decrease in viral prevalence in the second year of this study (2022–2023) compared to the first year (2021–2022), and only a 45% reduction in antibody prevalence during the same time. If neutralizing antibodies begin to wane over time in cervid populations, we might expect to see a resulting increase in viral prevalence. Experimental infections in deer would help determine if previous SARS-CoV-2 exposure provides immunity to homologous or heterologous variants.

Increasing immunity in cervids, combined with the decrease in SARS-Cov-2 prevalence in human populations compared to early in the pandemic, are likely the primary drivers in the decrease of SARS-CoV-2 in cervids that occurred during the course of this study. Experimental work has also shown early Omicron variants (B.1.1.529) to be less likely to infect transgenic mice and caused less disease in hamsters compared to previous variants^19^. We identified only 54 Omicron infections in deer out of 453 variants identified here, despite Omicron being the dominant variant in human populations during this study. If human populations continue to be infected with SARS-CoV-2 variants that appear to be highly human-adapted–possibly at the cost of efficient infection in other species–cervid infections may not reach the high levels seen during the beginning of the pandemic. Regardless, the patterns demonstrate what will continue to be a shifting landscape in the dynamics of SARS-CoV-2 in cervids and highlight the importance of continued long-term monitoring.

While data presented here and elsewhere^5,6^ demonstrate repeated spillover of SARS-CoV-2 into cervids from human populations, onward transmission of the virus from deer-to-deer, or cervid-to cervid, is supported as well. Experimental infection of mule deer and elk with a Delta variant SARS-Cov-2 virus demonstrated that mule deer could be infected with the virus and can transmit to uninfected conspecifics. Elk did not shed infectious virus^7^. In addition, genetically similar Alpha variant viruses were detected in deer in Pennsylvania and Ohio more than a year after Alpha variant viruses were last detected in people in that area, suggesting deer-to-deer transmission can occur alongside spillover from people^20^. Understanding SARS-CoV-2 dynamics in other wildlife species is also warranted as both fox^21^ and mink^22^ have shown the ability to transmit the virus to conspecifics, although white-tailed deer infections rates are still the highest of any wildlife species.

The high neutralizing antibody prevalence (21.43%) findings in comparison to rRT-PCR positives (5.58%) is not a surprise given the longer time horizon for antibody detection. Long-term studies of SARS-CoV-2 in white-tailed deer suggest antibodies can persist up to 13 months in captive white-tailed deer^23,24^. For samples where both SARS-CoV-2 RNA and antibodies were detected, findings could be indicative of prior exposure or could suggest that seroconversion happened rapidly, overlapping with the period that RNA was still detectable in swabs. Both dynamics–prior exposure and rapid seroconversion–are likely occurring, as evidenced by Palmer et al. (2022), who showed that seroconversion often occurs within one week of infection, but with SARS-CoV-2 RNA still being detectable in some cases up to 21 days post-infection^25^.

The Omicron sVNT assay detected additional neutralizing antibodies. Nearly 8% of samples that tested positive for neutralizing antibodies were only detected with the Omicron sVNT assay. Almost 23% of Omicron detections tested positive with the Omicron sVNT assay but not the ancestral sVNT assay. Using the Omicron sVNT assay in addition to the ancestral sVNT assay helped detect additional neutralizing antibodies in samples when the ancestral assay result is negative.

Broad geographic collection of both rRT-PCR and antibody data from wild white-tailed deer was the suggested next step from Chandler et al. (2021) for national surveillance, and the implementation and resultant findings provide a fuller picture of these dynamics. Broadscale and comprehensive findings like those reported here can also inform downstream research working to identify the transmission pathways moving virus from humans to cervids and back again^8^. Understanding these drivers is the next step in identifying management actions that could lessen spillover from people to wildlife, as well as spillback into human populations.

## Supplementary Information

Below is the link to the electronic supplementary material.

Supplementary Material 1

## Data Availability

The datasets analyzed during the current study are available in the supplementary material.
